# Hospital Notice

**Published:** 1872-11

**Authors:** E. R. Barnes

**Affiliations:** Secretary of Staff, 35 W. Eagle St.


HOSPITAL NOTICE.—Applicants for the position of resident physician to the Buffalo General Hospital are hereby notified to apply to the Secretary of the Staff before the 22d of February, 1873, when they will be notified by him of the time and place of examination.

E. R. BARNES,
Secretary of Staff, 35 W. Eagle St.

